# A Comparative Analysis of Synonymous Codon Usage Bias Pattern in Human Albumin Superfamily

**DOI:** 10.1155/2014/639682

**Published:** 2014-02-20

**Authors:** Hoda Mirsafian, Adiratna Mat Ripen, Aarti Singh, Phaik Hwan Teo, Amir Feisal Merican, Saharuddin Bin Mohamad

**Affiliations:** ^1^Institute of Biological Sciences, Faculty of Science, University of Malaya, 50603 Kuala Lumpur, Malaysia; ^2^Allergy and Immunology Research Centre, Institute for Medical Research, Jalan Pahang, Kuala Lumpur, Malaysia; ^3^Crystal, Institute of Biological Sciences, Faculty of Science, University of Malaya, 50603 Kuala Lumpur, Malaysia

## Abstract

Synonymous codon usage bias is an inevitable phenomenon in organismic taxa across the three domains of life. Though the frequency of codon usage is not equal across species and within genome in the same species, the phenomenon is non random and is tissue-specific. Several factors such as GC content, nucleotide distribution, protein hydropathy, protein secondary structure, and translational selection are reported to contribute to codon usage preference. The synonymous codon usage patterns can be helpful in revealing the expression pattern of genes as well as the evolutionary relationship between the sequences. In this study, synonymous codon usage bias patterns were determined for the evolutionarily close proteins of albumin superfamily, namely, albumin, *α*-fetoprotein, afamin, and vitamin D-binding protein. Our study demonstrated that the genes of the four albumin superfamily members have low GC content and high values of effective number of codons (ENC) suggesting high expressivity of these genes and less bias in codon usage preferences. This study also provided evidence that the albumin superfamily members are not subjected to mutational selection pressure.

## 1. Introduction

Amino acids, the monomeric unit of proteins, are encoded by triplet of nucleotides called codons. Most of the amino acids have alternative codons which are known as synonymous codons. The frequencies with which these synonymous codons are used are unequal [1], some codons being used preferentially than others. Furthermore, Plotkin et al. [2] reported that codon usage is tissue-specific. The phenomenon of codon usage bias, which can be interpreted as an outcome of either mutational bias or translational selection, is an essential feature of most genomes across all the three domains of life [3]. The patterns of codon usage within the mammalian genomes are markedly different from other taxa. In mammals, the codon usage bias is found to be influenced by the variation in isochores (GC content) or variation in tRNA pool of the cell [4, 5]. The differences in codon usage or the variation in tRNA abundance can elicit varied responses to the environmental changes, in terms of regulation of translation mechanism and cell phenotype [6]. Urrutia and Hurst [7] reported that, in humans, the codon usage bias is positively related to gene expression but is inversely related to the rate of synonymous substitution. Several factors contribute to synonymous codon usage bias such as gene expression level, protein hydropathy, protein secondary structure, and translational selection [8–11]. Information on the synonymous codon usage pattern can provide significant insights pertaining to the prediction, classification, and molecular evolution of genes and design of highly expressed genes and cloning vectors [12]. It may be useful in better understanding of host-pathogen interactions as information on synonymous codon usages can reveal about the host-pathogen coevolution and adaptation of pathogens to specific hosts [13].

The evolutionarily close proteins of albumin superfamily are comprised of albumin (ALB), *α*-fetoprotein (AFP), vitamin D-binding protein (VDBP), and afamin (AFM). In human, the genes encoding these proteins are mapped to chromosome 4. These proteins are synthesized primarily and predominantly in liver but the expression pattern varies temporally. One common functional property amongst all the members of albumin superfamily is their tendency to serve as transporters to various cellular components, metabolites, and so forth. ALB, an abundant serum protein of MW of ~66 KDa, binds and transports a variety of ligands such as steroids, fatty acids, bilirubin, lysolecithin, prostaglandins, thyroid hormones, and drugs. In addition to this, ALB is known to be involved in various cellular functions including oxygen-free radicals scavenging, anticoagulation, and maintenance of physiological pH and oncotic pressure of the plasma [14]. AFP (MW ~67 KDa), a serum glycoprotein which is expressed at high levels by fetal liver and visceral yolk sac [15, 16], is critical for the female fertility rather than embryonic development [17]. VDBP or Gc globulin (MW ~58 KDa) is synthesized by various tissues, namely, liver, kidneys, gonads, and fat, and also by neutrophils [18]. Apart from binding and transporting vitamin D sterols, VDBP's physiological functions include scavenging of G-actin [19], macrophage activation [20], and enhancement of chemotactic activity of C5a and C5a des-Arg molecules [21, 22]. AFM or *α*-albumin (MW ~87 KDa) is synthesized by liver and brain capillary endothelial cells. It mediates the transport of *α*-tocopherol across the blood-brain barrier [23].

The members of albumin superfamily have been found to act as markers in various disease states in humans. AFP in maternal serum is an indicative of Down's syndrome and neural tube defects in the fetus [24, 25]. AFP levels are elevated in patients with high risk for hepatocellular carcinoma. In some patients, an increase in AFP levels manifests liver metastasis with gastric cancer and the condition is termed as *α*-fetoprotein producing gastric cancer (AFPGC) [26, 27]. VDBP may serve as a biomarker for vascular injury as predicted by proteomic identification [28]. AFM may act as a potential adjunct marker to cancer antigen 125 (CA125) for the diagnosis of ovarian cancer [29]. A vast array of research has been done on the members of albumin superfamily; however, so far, studies related to the usage of synonymous codon and the factors influencing the codon usage in this gene family have not been done. In this study, we applied bioinformatics approaches to elucidate the pattern of synonymous codon usage bias and its consequences on the expression level of genes in the albumin superfamily.

## 2. Materials and Methods

### 2.1. Sequences

The mRNA reference sequences of human serum albumin (ALB), afamin (AFM), *α*-fetoprotein (AFP), and vitamin D-binding protein (VDBP) in FASTA format were retrieved from GenBank of the National Center for Biotechnology Information (NCBI) (http://www.ncbi.nlm.nih.gov/genbank/). Open Reading Frame (ORF) of the mRNA sequences of human albumin superfamily was obtained by using ExPASy Translate tool (http://web.expasy.org/translate/).

### 2.2. Hydrophobicity Analysis

Grand average of hydrophobicity score (Gravy score) was calculated to quantify the general average hydrophobicity for the translated gene product found in albumin superfamily. It was calculated as the arithmetic mean of the sum of the hydrophobic indices of each amino acid as shown in

(1) $$\begin{array}{c} \frac{1}{N}\sum_{i=1}^{N}K_{i}, \end{array}$$

where *N* corresponds to the number of amino acids, while *K*_*i*_ represents hydrophobic index of amino acid. The Gravy score of a protein can be either negative or positive depending on the frequency of amino acids with distinct properties. Negative Gravy score implies that the protein is hydrophilic and is soluble in water. In contrast, protein with positive Gravy is considered as hydrophobic and is water soluble [30].

### 2.3. Codon Usage Analysis

The nucleotide distribution for albumin superfamily was analyzed using ExPASy ProtParam tool (http://web.expasy.org/protparam/). The quantities of individual nucleotide (A, T, G, and C) were determined and used to sum up the AT and GC content for each protein in the albumin superfamily.

### 2.4. Rare Codon (RC) Analysis

Rare codon (RC) is considered as low-usage codon in the genome such as synonymous codon or stop codon [31]. The RC analysis was performed using the GenScript web server (http://www.genscript.com/cgi-bin/tools/rare_codon_analysis/) to examine the number of highest-usage and lowest-usage codons in the human albumin superfamily.

### 2.5. Indices of Codon Usage Deviation

Indices of codon usage deviation were calculated using CodonW (J Peden, version 1.4.2 http://codonw.sourceforge.net/) [32] to measure deviation between the observed codon usage and expected codon usage. Based on that, two internal measures were applied including identification of GC variation and third nucleotide preference in codon [33, 34]. These were obtained by calculating the number of GC nucleotides and number of G or C nucleotides at the third position of synonymous codon (GC_3_), except the start and termination codons. In addition, the expected effective number of codons (ENC) for each albumin superfamily protein was calculated. ENC is the measure of codon usage affected only by the GC_3_ as a consequence of mutation pressure and genetic drift. The ENC was calculated according to [35]

(2) $$\begin{array}{c} \text{ENC}=2+s+\frac{29}{s^{2}+{\left(1-s\right)}^{2}}, \end{array}$$

where *s* corresponds to the GC_3_ value ranging from 0 to 100%.

### 2.6. Relative Synonymous Codon Usage (RSCU)

Relative synonymous codon usage (RSCU) was calculated in order to examine the frequency of each synonymous codon that encoded the same amino acid without confounding effect on the composition of amino acid. The index was calculated as follows [36]:

(3) $$\begin{array}{c} \text{RSC}{\text{U}}_{ij}=\frac{X_{ij}}{\left(1/n_{i}\right)\sum_{j=1}^{n_{i}}X_{ij}\;}, \end{array}$$

where *X*_*ij*_ is the amount of *j*th codon to represent the *i*th amino acid that can be encoded by *n*_*i*_ synonymous codons.

## 3. Results and Discussion

**Figure 1:**
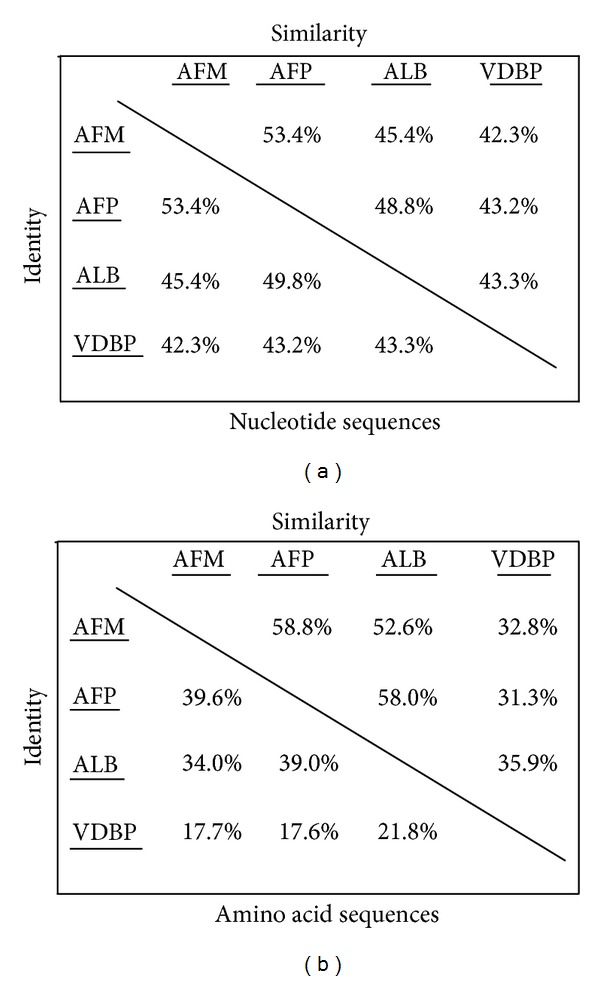
Comparison of percent similarity and identity of nucleotide sequences and amino acid sequences of human albumin superfamily members.

Genomic information of mRNA sequences of the four members of human albumin superfamilyis shown in Table 1. The mRNA sequences of albumin superfamily were translated into protein sequences using the ExPASy Translate Tool. Only the ORF with no intermediate stop codon was selected for codon usage analysis. The similarity of nucleotide and amino acid sequences of the albumin superfamily members is summarized in Figure 1. The results showed that ALB and AFP are more closely related compared to AFM and VDBP. AFP and VDBP have almost similar gene length of 2032 bp and 2024 bp, respectively. ALB possesses the longest (2264 bp), while AFM has the shortest gene length (1997 bp). Moreover, human ALB and AFP possessed exactly the same length of ORF (1830 bp), while AFM (1800 bp) has similar length of the ORF compared to that of ALB and AFP. VDBP (1425 bp) has the shortest length of ORF within the albumin superfamily. The similarity pattern of ORF among ALB, AFM, and AFP indicated that they may carry out similar biological functions, especially AFM, since its function is not well-known.

The solubility of protein for the members of the albumin superfamily was assessed through Gravy score (Table 1). All the family members are found to have negative Gravy score, suggesting that these proteins are water soluble. This is in accordancewith the biological role of these proteins as serum transporters.

The nucleotide distribution of albumin superfamily is shown in Table 2. The members of this superfamily exhibit low GC content (<44.63%). ALB and AFP shows similar nucleotide distribution pattern implying that they share similarity in their structures and biological functions. There is a close relationship between the nucleotide composition and gene function [37]. AFM has the highest AT content, whereas VDBP has the lowest AT content. Although AFM and VDBP are grouped in the same superfamily, they show differential nucleotide composition suggesting variation in their biological functions compared to the other members of albumin superfamily.

**Figure 2:**
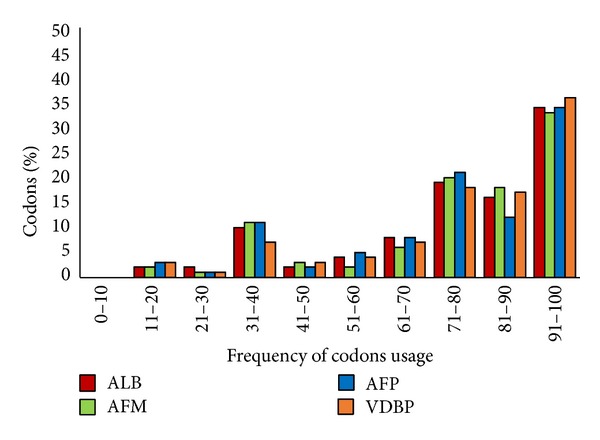
Codon frequency distribution of human albumin superfamily members.

Rare codon analysis was carried out using the GenScript web server as described in Materials and Methods. A graph of codon frequency distribution was plotted to identify the quantities of rare codons present in each albumin superfamily protein (Figure 2). Frequency of codon usage with a value of 100 indicates that the codons are highly used for a given amino acid. Conversely, the frequency of codon usage with a value of less than 30 is determined as low-frequency codon, which is likely to affect the expression efficiency. Percentages of low-frequency codon present in protein ALB, AFM, AFP, and VDBP are 4%, 3%, 4%, and 4%, respectively. This result suggested that members of the albumin superfamily contain a significantly small number of rare codons that may reduce translational efficiency of the genes.

Indices of codon usage deviation are used to determine the differences between the observed and expected codon usage. The results for the effective number of codon (ENC), GC content, and G or C nucleotides at the third position of synonymous codon are summarized in Table 1. The effective number of codons (ENC) for each member of human albumin superfamily was calculated in order to examine the pattern of synonymous codon usage independent of the gene length. The ENC value ranges from 20 to 61, in which value of 20 indicates extreme bias toward the usage of one codon, while value of 61 represents equal usage of the synonymous codons [35, 38]. Result from this analysis revealed that the ENC value of albumin superfamily varies from 51.65 to 56.62. The overall ENC value of albumin superfamily is greater than 50. The high ENC value suggested that the synonymous codons of albumin superfamily were equally used and hence displayed less biased synonymous codon usage.

The GC content of albumin superfamily is given in Table 1. GC content may affect the thermostability, bendability, and the ability of DNA helix transition from B to Z form. GC content can be related to the ability of coding region to be in an open chromatin state, leading to active transcription [39]. It is evident that all the members of albumin superfamily genes have low GC content, indicating that these family members are highly expressed. Furthermore, it has been reported that highly transcribed genes may have low mutation rates because they are subjected to DNA repair [40]. However, within the albumin superfamily, VDBP contains the highest GC content indicating that it has the lowest expressivity level.

GC content at the third position of codons (GC_3_) is a putative indicator of the extent of base composition bias. Table 1 revealed that the albumin superfamily has low GC_3_ values ranging from 37.1% to 42.8%. The albumin superfamily has low GC_3_ value because the majority of genes in this superfamily are located in AT-rich region. Genes in AT-rich regions within the genome would prefer to use A or T ending codon. The low usage of codons ending with G or C signifies less GC codon usage bias in albumin superfamily. In other words, it proved the homogeneity of synonymous codon usage pattern in albumin superfamily.

The synonymous codon bias usage of each albumin superfamily protein was computed and tabulated in Table 3. The most preferentially used codon for a given amino acid is highlighted in red. Asn of AFP and His, Cys, and Arg of VDBP have equal usage of the synonymous codons. The variation of relative synonymous codon usage (RSCU) values not only indicated the different frequency of occurrence of each codon for a given amino acid in different albumin superfamily protein but also revealed the preference of either A + U or G + C codon usage as listed in Table 3. The results of RSCU analysis (Table 3) are summarized in Table 4. Preferential codon usage in albumin superfamily indicates that the codons with A or U at the third position are more preferred compared to G or C ending codons. Table 4 also shows that the total score of A + U and G + C codon usage in the proteins of albumin superfamily is not equal to 20. It is because some amino acid residues are encoded in equal frequencies by both A or U and G or C ending codons and hence are excluded from the analysis. The tendency of albumin superfamily to use high A + U and low G + C indicated that the mutational bias does not play a significant role in synonymous codon usage.

## 4. Conclusions

The members of albumin superfamily, namely, ALB, AFP, AFM, and VDBP, exhibit sequence and structural similarities. The proteins possess three homologous folding domains as a result of conserved pattern of cysteine residues in the members of albumin superfamily [41, 42]. Our study on codon usage bias in the members of the albumin gene family revealed that they are also similar in terms of their low GC content, low GC_3_, and high ENC values. In addition, they are not having a bias in the usage of synonymous codons and are highly expressible genes. Furthermore, low GC and GC_3_ values revealed that mutational bias and translational selection do not play a significant role in shaping the codon usage pattern in the albumin superfamily.

## Figures and Tables

**Table 1:** Genomic information of the reference sequences, grand average hydrophobicity score, ENCs, GC content, and GC_3_ of human albumin superfamily members.

	Human albumin superfamily			
	Albumin (ALB)	Afamin (AFM)	Alpha-fetoprotein (AFP)	Vitamin D-binding protein (VDBP)
GenBank accession number	NM_000477.5	NM_001133.2	NM_001134.1	NM_000583.3
Gene length (bp)	2264	1997	2032	2024
Grand average of hydrophobicity score (Gravy score)	−0.354	−0.248	−0.388	−0.336
GC content	42.95	42.02	39.28	44.63
Effective number of codons (ENC)	53.91	51.65	54.78	56.62
GC_3_	38.00	37.10	37.30	42.80

**Table 2:** Nucleotide distribution of human albumin superfamily members.

	ALB (%)	AFP (%)	AFM (%)	VDBP (%)
A	30.4 (556)	32.6 (596)	32.8 (591)	29.9 (426)
T	26.7 (488)	25.4 (465)	27.9 (502)	25.5 (363)
G	23.0 (421)	21.7 (397)	20.1 (361)	21.4 (305)
C	19.9 (365)	20.3 (372)	19.2 (346)	23.2 (331)
AT	57.049	57.978	60.722	55.368
GC	42.951	42.022	39.278	44.632

The values in parenthesis represent the number of individual nucleotides in the genes of human albumin superfamily members.

**Table 3:** Relative synonymous codon usage in human albumin superfamily members. The value in bold indicates the codons used with high frequency.

Amino acid	Codons	RSCU^1^	Number	RSCU^2^	Number	RSCU^3^	Number	RSCU^4^	Number
Phe	UUU	**1.43**	25	**1.06**	17	**1.30**	28	**1.16**	11
	UUC	0.57	10	0.94	15	0.70	15	0.84	8

Leu	UUA	0.94	10	1.00	10	**1.20**	11	0.53	5
	UUG	1.13	12	1.10	11	0.87	8	0.63	6
	CUU	**1.78**	19	0.90	9	**1.20**	11	1.16	11
	CUC	0.66	7	0.40	4	0.98	9	0.95	9
	CUA	0.38	4	0.90	9	0.65	6	1.05	10
	CUG	1.13	12	**1.70**	17	1.09	10	**1.68**	16

Ile	AUU	**1.33**	4	**1.32**	15	1.07	10	**1.13**	3
	AUC	**1.33**	4	0.71	8	0.75	7	**1.13**	3
	AUA	0.33	1	0.97	11	**1.18**	11	0.75	2

Val	GUU	1.12	12	**1.47**	11	**1.44**	13	0.89	6
	GUC	0.65	7	0.80	6	0.67	6	**1.19**	8
	GUA	0.74	8	0.80	6	0.67	6	1.04	7
	GUG	**1.49**	16	0.93	7	1.22	11	0.89	6

Ser	UCU	0.64	3	1.26	8	**2.06**	12	1.43	10
	UCC	**1.50**	7	0.47	3	0.86	5	1.29	9
	UCA	1.29	6	**1.58**	10	1.03	6	**1.71**	12
	UCG	0.64	3	0.47	3	0.00	0	0.00	0

Pro	CCU	**1.67**	10	**1.71**	9	**1.71**	12	1.38	9
	CCC	1.00	6	0.76	4	0.71	5	0.92	6
	CCA	1.17	7	1.52	8	1.57	11	**1.54**	10
	CCG	0.17	1	0.00	0	0.00	0	0.15	1

Thr	ACU	0.97	7	**1.78**	16	1.18	10	1.25	10
	ACC	1.24	9	0.67	6	0.82	7	**1.38**	11
	ACA	**1.52**	11	1.33	12	**1.53**	13	1.13	9
	ACG	0.28	2	0.22	2	0.47	4	0.25	2

Ala	GCU	**1.90**	30	1.20	15	**1.57**	11	**1.82**	15
	GCC	0.89	14	0.88	11	0.71	5	1.09	9
	GCA	1.08	17	**1.68**	21	1.29	9	0.85	7
	GCG	0.13	2	0.24	3	0.43	3	0.24	2

Tyr	UAU	**1.37**	13	**1.06**	9	**1.06**	9	**1.13**	9
	UAC	0.63	6	0.94	8	0.94	8	0.88	7

His	CAU	**1.38**	11	**1.63**	13	**1.23**	8	**1.00**	4
	CAC	0.63	5	0.38	3	0.77	5	**1.00**	4

Gln	CAA	**1.10**	11	**1.15**	23	**1.26**	17	**1.33**	8
	CAG	0.90	9	0.85	17	0.74	10	0.67	4

Asn	AAU	**1.29**	11	**1.00**	10	**1.03**	17	**1.33**	12
	AAC	0.71	6	**1.00**	10	0.97	16	0.67	6

Lys	AAA	**1.33**	40	**1.29**	33	**1.33**	28	0.93	20
	AAG	0.67	20	0.71	18	0.67	14	**1.07**	23

Asp	GAU	**1.39**	25	**1.27**	21	**1.30**	15	**1.23**	16
	GAC	0.61	11	0.73	12	0.70	8	0.77	10

Glu	GAA	**1.23**	38	**1.24**	34	**1.36**	40	**1.26**	27
	GAG	0.77	24	0.76	21	0.64	19	0.74	16

Cys	UGU	0.86	15	**1.06**	18	0.81	13	**1.00**	14
	UGC	**1.14**	20	0.94	16	**1.19**	19	**1.00**	14

Arg	CGU	**0.67**	3	**0.50**	2	**0.55**	2	0.00	0
	CGC	0.22	1	0.25	1	0.27	1	0.00	0
	CGA	**0.67**	3	**0.50**	2	**0.55**	2	**0.92**	2
	CGG	0.44	2	0.25	1	0.00	0	0.46	1

Ser	AGU	**1.29**	6	**1.54**	9	0.79	5	**0.86**	6
	AGC	0.64	3	0.51	3	**1.42**	9	0.71	5

Arg	AGA	**2.89**	13	**2.75**	11	**3.27**	12	**2.31**	5
	AGG	1.11	5	1.75	7	1.36	5	**2.31**	5

Gly	GGU	0.92	3	0.75	3	0.62	4	0.29	1
	GGC	0.92	3	0.75	3	0.77	5	**1.43**	5
	GGA	**1.85**	6	**1.50**	6	**2.00**	13	**1.43**	5
	GGG	0.31	1	**1.50**	4	0.62	4	0.86	3

RSCU^1 ^: RSCU values for ALB; RSCU^2^: RSCU values for AFP; RSCU^3^: RSCU values for AFM; RSCU^4^: RSCU values for DBP.

**Table 4:** A + U and G + C preferential codon usage of human albumin superfamily members.

	A + U	G + C
ALB	17	3
AFP	17	1
AFM	18	2
VDBP	11	4

## References

[1] Bennetzen JL, Hall BD (1982). Codon selection in yeast. *Journal of Biological Chemistry*.

[2] Plotkin JB, Robins H, Levine AJ (2004). Tissue-specific codon usage and the expression of human genes. *Proceedings of the National Academy of Sciences of the United States of America*.

[3] Duret L (2002). Evolution of synonymous codon usage in metazoans. *Current Opinion in Genetics and Development*.

[4] Bernardi G, Olofsson B, Filipski J (1985). The mosaic genome of warm-blooded vertebrates. *Science*.

[5] Dittmar KA, Goodenbour JM, Pan T (2006). Tissue-specific differences in human transfer RNA expression. *PLoS Genetics*.

[6] Najafabadi HS, Goodarzi H, Salavati R (2009). Universal function-specificity of codon usage. *Nucleic Acids Research*.

[7] Urrutia AO, Hurst LD (2001). Codon usage bias covaries with expression breadth and the rate of synonymous evolution in humans, but this is not evidence for selection. *Genetics*.

[8] Gupta SK, Ghosh TC (2001). Gene expressivity is the main factor in dictating the codon usage variation among the genes in *Pseudomonas aeruginosa*. *Gene*.

[9] Liu Q (2006). Analysis of codon usage pattern in the radioresistant bacterium *Deinococcus radiodurans*. *BioSystems*.

[10] D’Onofrio G, Ghosh TC, Bernardi G (2002). The base composition of the human genes is correlated with the secondary structures of the encoded proteins. *Gene*.

[11] Naya H, Romero H, Carels N, Zavala A, Musto H (2001). Translational selection shapes codon usage in the GC-rich genome of *Chlamydomonas reinhardtii*. *FEBS Letters*.

[12] Liu X-S, Zhang Y-G, Fang Y-Z, Wang Y-L (2012). Patterns and influencing factor of synonymous codon usage in porcine circovirus. *Virology Journal*.

[13] Pandit A, Sinha S (2011). Differential trends in the codon usage patterns in HIV-1 genes. *PLoS ONE*.

[14] Nicholson JP, Wolmarans MR, Park GR (2000). The role of albumin in critical illness. *British Journal of Anaesthesia*.

[15] Meehan RR, Barlow DP, Hill RE, Hogan BL, Hastie ND (1984). Pattern of serum protein gene expression in mouse visceral yolk sac and foetal liver. *The EMBO Journal*.

[16] Dziadek MA, Andrews GK (1983). Tissue specificity of alpha-fetoprotein messenger RNA expression during mouse embryogenesis. *The EMBO Journal*.

[17] Gabant P, Forrester L, Nichols J (2002). Alpha-fetoprotein, the major fetal serum protein, is not essential for embryonic development but is required for female fertility. *Proceedings of the National Academy of Sciences of the United States of America*.

[18] Chishimba L, Thickett DR, Stockley RA, Wood AM (2010). The vitamin D axis in the lung: a key role for vitamin D-binding protein. *Thorax*.

[19] Otterbein LR, Cosio C, Graceffa P, Dominguez R (2002). Crystal structures of the vitamin D-binding protein and its complex with actin: structural basis of the actin-scavenger system. *Proceedings of the National Academy of Sciences of the United States of America*.

[20] Mohamad SB, Nagasawa H, Uto Y, Hori H (2002). Preparation of Gc protein-derived macrophage activating factor (GcMAF) and its structural characterization and biological activities. *Anticancer Research*.

[21] Kew RR, Webster RO (1988). Gc-globulin (vitamin D-binding protein) enhances the neutrophil chemotactic activity of C5a and C5a des Arg. *Journal of Clinical Investigation*.

[22] DiMartino SJ, Shah AB, Trujillo G, Kew RR (2001). Elastase controls the binding of the vitamin D-binding protein (Gc-Globulin) to neutrophils: a potential role in the regulation of C5a co-chemotactic activity. *Journal of Immunology*.

[23] Kratzer I, Bernhart E, Wintersperger A (2009). Afamin is synthesized by cerebrovascular endothelial cells and mediates *α*-tocopherol transport across an in vitro model of the blood-brain barrier. *Journal of Neurochemistry*.

[24] Seller MJ (1990). Alphafetoprotein in midtrimester Down’s syndrome fetal serum. *Journal of Medical Genetics*.

[25] Brock JH (1976). Alphafetoprotein and neural tube defects. *Journal of Clinical Pathology*.

[26] Hirajima S, Komatsu S, Ichikawa D (2013). Liver metastasis is the only independent prognostic factor in AFP-producing gastric cancer. *World Journal of Gastroentrology*.

[27] Li X-D, Wu C-P, Ji M (2013). characteristic analysis of a-fetoprotein-producing gastric carcinoma in China. *World Journal of Surgical Oncology*.

[28] Huang NF, Kurpinski K, Fang Q, Lee RJ, Li S (2011). Proteomic identification of biomarkers of vascular injury. *The American Journal of Translational Research*.

[29] Dieplinger H, Ankerst DP, Burges A (2009). Afamin and apolipoprotein A-IV: novel protein markers for ovarian cancer. *Cancer Epidemiology Biomarkers and Prevention*.

[30] Kyte J, Doolittle RF (1982). A simple method for displaying the hydropathic character of a protein. *Journal of Molecular Biology*.

[31] Ikemura T (1981). Correlation between the abundance of Escherichia coli transfer RNAs and the occurrence of the respective codons in its protein genes: a proposal for a synonymous codon choice that is optimal for the *E. coli* translational system. *Journal of Molecular Biology*.

[32] Peden JF (2000). *Analysis of Codon Usage*.

[33] Novembre JA (2002). Accounting for background nucleotide composition when measuring codon usage bias. *Molecular Biology and Evolution*.

[34] Comeron JM, Aguadé M (1998). An evaluation of measures of synonymous codon usage bias. *Journal of Molecular Evolution*.

[35] Wright F (1990). The ’effective number of codons’ used in a gene. *Gene*.

[36] Nei M, Kumar S (2000). *Molecular Evolution and Phylogenetics*.

[37] Garcia JAL, Fernández-Guerra A, Casamayor EO (2011). A close relationship between primary nucleotides sequence structure and the composition of functional genes in the genome of prokaryotes. *Molecular Phylogenetics and Evolution*.

[38] Fuglsang A (2004). The effective number of codons. *Biochemical and Biophysical Research Communications*.

[39] Vinogradov AE (2003). DNA helix: the importance of being GC-rich. *Nucleic Acids Research*.

[40] Berg OG, Martelius M (1995). Synonymous substitution-rate constants in *Escherichia coli* and *Salmonella typhimurium* and their relationship to gene expression and selection pressure. *Journal of Molecular Evolution*.

[41] Lichenstein HS, Lyons DE, Wurfel MM (1994). Afamin is a new member of the albumin, *α*-fetoprotein, and vitamin D-binding protein gene family. *Journal of Biological Chemistry*.

[42] Nishio H, Dugaiczyk A (1996). Complete structure of the human *α*-albumin gene, a new member of the serum albumin multigene family. *Proceedings of the National Academy of Sciences of the United States of America*.

